# Changes in the gut microbiome of older adults according to hypertension control

**DOI:** 10.3389/fmicb.2025.1605271

**Published:** 2025-09-02

**Authors:** Fernanda Valdez-Palomares, Tomas Texis, Sergio Sánchez-García, José Darío Martínez-Ezquerro, Paola García-de la Torre, Mauricio Rodríguez-Dorantes, Alma Genis-Mendoza, Salvador Fabela, Berenice Palacios-González, Vanessa González-Covarrubias

**Affiliations:** ^1^Instituto Nacional de Medicina Genómica (INMEGEN), CDMX, Mexico City, Mexico; ^2^Centro de Investigación Sobre Envejecimiento (CIE-CINVESTAV Sur), CDMX, Mexico City, Mexico; ^3^Unidad de Investigación Epidemiológica y en Servicios de Salud, Área Envejecimiento, Centro Médico Nacional Siglo XXI, Instituto Nacional del Seguro Social (IMSS), CDMX, Mexico City, Mexico; ^4^Hospital Psiquiátrico Infantil Juan N. Navarro, CDMX, Mexico City, Mexico

**Keywords:** older adults, gut microbiome, hypertension, diastolic blood pressure, systolic blood pressure

## Abstract

Hypertension is the leading cause of cardiovascular disease, with over 60% prevalence in older adults, and its control is complex and requires multidisciplinary approaches. The role of the gut microbiome in blood pressure control remains unclear despite reported associations of some specific bacteria involved in the development of hypertension. The aim of this study was to characterize the gut microbiome of older adults and to identify bacteria associated with hypertension control. Patients aged 60 years and older from Mexico City and the metropolitan area, all of whom were receiving antihypertensive treatment, provided a feces sample during a routine medical visit. DNA was extracted from 240 samples using a commercial kit, the V3/V4 region of the 16S gene was sequenced, and metataxonomic analyses were performed using QIIME and R. Bacterial abundance analysis identified a core microbiome in the hypertensive older adults, with an increased abundance of *Escherichia-Shigella* and a decrease in alpha diversity with increasing age. *Ruminococcus UCG-002*, *DTU 089,* and members of the *Lachnospiraceae* family were distinctively abundant in controlled hypertension. These bacteria are fiber-fermenting and producers of short-chain fatty acids (SCFAs), and their differential abundance according to hypertension control suggests an intricate interplay among SCFA producers. Our results confirm and expand upon previous reports on the core gut microbiome of older adults, suggesting relevant changes in fiber-fermenting bacteria—*Ruminococcus UCG-002*, *DTU 089,* and members of the *Lachnospiraceae* family—for hypertension control.

## 1 Introduction

The study of the gut microbiome has shown a significant impact on the understanding of the development, progression, and control of blood pressure. The production of short-chain fatty acids (SCFAs) by the gut microbiota seems to be crucial for the activation of key receptors that can regulate blood pressure in different directions. Several studies have confirmed the link between the gut microbiome and cardiovascular diseases, with some studies establishing causative relationships (Dong et al., 2013; Yang et al., 2015). In a cohort study, Sun et al. established a connection between the gut microbiome and hypertension, observing decreased microbial diversity with specific microorganisms associated with high blood pressure and revealing a compromised gut barrier, gut dysbiosis, and inflammation (Sun et al., 2019).

Although genetics, environment, diet, and the gut microbiome are crucial to the development of hypertension, substantial evidence suggests that age plays a major role, with up to 65% of older adults being hypertensive in many regions of the world. Even so, there is an apparent decline in the gut microbiome’s diversity and richness after 60 years of age (Haran and McCormick, 2021). However, the definitive characteristics of the gut microbiome in older adults have only been studied in certain populations (Zapata and Quagliarello, 2015), and it needs to be more comprehensively investigated (Althani et al., 2016; Al Khodor et al., 2017).

More recently, the scientific community has uncovered that gut bacteria can affect the pharmacokinetics and pharmacodynamics of antihypertensive medications through metabolic enzymes that can reduce drug bioavailability prior to drug absorption (Zimmermann et al., 2019). In addition, antihypertensive drugs can alter the gut microbiome’s composition. Yang T et al. observed an enrichment of the *Coprococcus* genus in patients with a poor response to ACE inhibitors, which differed by geographical ancestry (Yang et al., 2022). Similarly, the reduction of systolic blood pressure (SBP) after captopril and losartan administration reduces gut dysbiosis in hypertensive rats (Robles-Vera et al., 2020), while diuretics combined with beta blockers and ACE inhibitors have been associated with the enrichment of *Roseburia* (Forslund et al., 2021).

Hypertension control is key to reducing cardiovascular mortality, the leading cause of death worldwide. However, the complexity of diagnosing and managing hypertension contributes to its high prevalence, despite the availability of over 65 different antihypertensive drugs. Most of the current investigations have defined the role of the gut microbiome in hypertension by comparing patients with normotensive individuals, and little is known about the impact of hypertension control on the gut microbiome and its potential benefits. Moreover, the identification of microbes influencing blood pressure has accumulated information for certain populations, but the high variability and the apparent influence of environment and genetics highlight the importance of validating these associations in larger study groups from different geographic ancestries. Here, we describe the diversity and abundance of the gut microbiome in admixed older adults, focusing on hypertension control.

## 2 Materials and methods

### 2.1 Study population

Participants (*N* = 240) aged over 60 years were invited to take part by donating a fecal sample between 2017 and 2022 at the Hospital Centro Medico Nacional Siglo XXI (CMN-IMSS), All participants signed an informed consent form. The inclusion criteria consisted of male and female individuals aged 60 years and older, diagnosed with hypertension, and receiving antihypertensive treatment for at least 4 years at the time of recruitment. The exclusion criteria included diagnosis of cancer, chronic neurodegenerative or immune diseases, and insufficient or deficient sample quality. The protocol was approved by the Committees of Research Ethics under approval numbers R2018-785-004 and CEI2017/04 & 23/2016/I. This research followed current bioethical and safety regulations, including the principles of the Declaration of Helsinki. Fecal samples were collected from patients who were carefully instructed and provided with an in-house collection kit. Samples were added in RNA-later (Thermo-Scientific) and stored at −70°C until DNA extraction. Blood pressure was measured three times within 15–30 min using a sphygmomanometer. The patients were then classified as controlled (<140/90 mmHg) and uncontrolled (≥140/90 mmHg) according to institutional guidelines. This study focused on comparing these groups based on all available clinical and demographic data; however, it did not include a normotensive group or its lifestyle habits.

### 2.2 DNA extraction and 16S rRNA V3/V4 sequencing

DNA was isolated from 200 mg of feces using the QIAamp Fast DNA Stool Mini Kit (Qiagen, United States) according to the manufacturer’s instructions and stored at −20°C. The hypervariable region V3-V4 was amplified using the 16S V3 (341F) forward and V4 (805R) reverse primers and adapters from Illumina following the manufacturer’s 16S metagenomic sequencing library protocol. PCR reactions were 30 μL in volume, containing 4 μL of the DNA (50 ng/μL), 0.25 μL of each PCR primer (10 pM), and 15 μL of 2X Platinum™ SuperFi™ PCR Master Mix (Invitrogen, United States). Amplification was performed for 25 cycles consisting of 95°C for 30 s, 55°C for 30 s, and 72°C for 30 s, followed by a final extension at 72°C for 5 min. The fragments were cleaned with Agencourt AMPure XP beads (Beckman Coulter Genomics, Brea, CA, United States) according to the manufacturer’s protocol. Indexes and adaptors were ligated by PCR with 5 μL of Illumina Nextera XT Index Primer 1 (N7XX), 5 μL of Nextera XT Index Primer 2 (S5XX), and 25 μL of 2X Platinum™ SuperFi™ PCR Master Mix (Invitrogen, USA) in a thermocycler at 95°C for 3 min, as well as six cycles at 95°C for 30 s, 55°C for 30 s, and 72°C for 30 s, and a final extension at 72°C for 5 min. The 16S rRNA V3-V4 libraries were purified with Agencourt AMPure XP beads. Library quality control was verified by microcapillary electrophoresis using a TapeStation 4,200 (Agilent Technologies, CA, United States). Then, the libraries were normalized and pooled to 10.2 nM, denatured, and diluted to a final concentration of 10 pM, including 20% of PhiX. The libraries were sequenced using a 2x250bp cartridge/MiSeq Reagent Kit V3 in a MiSeq sequencer (Illumina).

### 2.3 Bioinformatic analyses

Sequencing paired-end FASTQ files were evaluated for quality control using QIIME2 v2024.5, followed by denoising with the Divisive Amplicon Denoising Algorithm 2 (DADA2) plugin. The resulting amplicon sequence variants (ASVs) were used to generate a taxonomy table with a naive Bayes pre-trained classifier for the V3-V4 hypervariable region of the 16S rRNA gene, based on the ribosomal database SILVA_138. QIIME2 artifacts were imported into R using the qiime2R package and analyzed with the Phyloseq package (Hall and Beiko, 2018). Statistical analyses were conducted in R version 4.0.4 (R Core Team, 2015).

To assess batch effects and batch correction, we conducted principal coordinate analysis (PCA) on adjusted and unadjusted rarefied relative abundance data with centered log-ratio normalization using the microViz R library. Rarefaction was set to the minimum sampling depth across samples, which was 26,500 sequences per sample. The core microbiome was assessed based on a sample prevalence of > 50% at a relative abundance frequency of > 1% at the genus level. Several alpha diversity indexes were assessed, including the observed species, Shannon index, Chao index, Simpson dissimilarity, and Fisher index. Significant differences in alpha diversity across the groups were calculated using the Kruskal–Wallis and Wilcoxon tests. PCA and redundancy analysis (RDA) were conducted with centered log-ratio normalized counts at the genus level using the microViz R library. The RDA included clinical variables, such as age, uncontrolled systolic or diastolic blood pressure (DBP), cholesterol, HDLC, LDLC, glucose, and triglycerides. Beta diversity was calculated using Bray–Curtis dissimilarity distances. In addition, a permutational multivariate analysis of variance (PERMANOVA) was conducted on 999 permutations to test the association between the composition of the microbiota and clinical variables, such as SBP, DBP, age, and sex. Differential abundance analyses at the genus level were performed using the linear regression framework for differential abundance analysis (LinDA), fitting a linear model for abundance data and correcting for compositional effects and biases. *p*-values were adjusted using the false discovery rate (FDR) method, with a significance threshold set at a *p*-value of ≤ 0.01.

In addition, we investigated hypertension-linked bacterial taxa by performing linear regression analyses of bacterial abundance and blood pressure control. Bacteria abundances were CLR-transformed to account for compositionality, and the models were adjusted for age, sex, and diabetes status. Significance was determined at a *p*-value of < 0.05, with effect sizes reported as *β*-coefficients, reflecting blood pressure changes in mmHg per unit increase in CLR-transformed abundance. These analyses specifically tested whether previously reported hypertension-associated taxa showed consistent relationships with blood pressure gradients in our cohort, irrespective of clinical control status.

Finally, functional prediction and differential abundance analysis were performed to predict functional profiles from ASVs using PICRUSt2 (v2.5.0). Predictions were reported as enzyme commission (EC) numbers and KEGG orthologs (KOs). Functional predictions were analyzed using DESeq2 (v1.40.0), comparing uncontrolled (SBP ≥ 140 mmHg or DBP ≥ 90 mmHg) versus controlled (SBP < 140 mmHg or DBP < 90 mmHg) groups. Features with a log2FC of > 1 and an FDR-adjusted *p*-value of < 0.05 were considered significant. Data were normalized using a variance-stabilizing transformation (VST). Confounding factors, such as age, sex, and T2D status, were included as covariates in the DESeq2 model. Analyses were performed separately for EC numbers and KOs to identify hypertension-associated metabolic shifts.

## 3 Results

We investigated the gut microbiome in 240 patients—113 male and 127 female individuals—aged between 60 and 95 years, all of whom had been receiving antihypertensive treatment for at least 4 years. Table 1 presents the demographic, clinical, and pharmacological characteristics of the study population. In addition to antihypertensive treatment, the most commonly prescribed drugs were lipid-lowering medication (36%), proton pump inhibitors (21%), antidiabetics (metformin or sulfonylureas, 50%), and NSAIDs (43%). Blood lipid levels showed significant differences between the male and female participants, as reported elsewhere. The individuals were classified by age group: 60–70y (69%), 71–80y (30%), and >80y (8.3%). They were also categorized according to systolic and diastolic blood pressure control as controlled (<140/90 mmHg) and uncontrolled (≥140/90 mmHg; Table 1).

**Table 1:** Study population characteristics.

Characteristic	All *N* = 240	Male *N* = 113 (47%)	Female *N* = 127 (53%)
Age (y)	68 (60–95)	68 (60–95)	68 (60–95)
Height, m	1.5 (1.37–1.79)	1.65 (1.44–1.79)	1.52 (1.37–1.65)
Weight, kg	71.7 (45–124)	77.0 (50.6–124)	66.8 (45–116)
SBP (mmHg)	130 (79–217)	131 (94–188)	130 (79–217)
DPB (mmHg)*	74 (47–134)	77 (47–107)	71 (52–134)
Glucose (mg/dL)	103 (48–780)	104 (48–780)	103 (60–426)
Triglycerides (mg/dL)	164 (52–780)	164 (52–582)	163 (52–780)
Cholesterol (mg/dL)*	186 (64–576)	164 (64–576)	203 (100–318)
HDL-C (mg/dL)*	47 (21–153)	41 (21–153)	52 (26–102)
LDL-C (mg/dL)*	105 (24–392)	101 (24–392)	110 (44–215)
Antihypertensive drugs	1 (1–5)	1 (1–5)	1 (1–4)
Other drugs	1 (0–6)	1 (0–6)	1 (0–6)

Data are mean values, with ranges in the parentheses. Controlled SBP < 140 mmHg, mean 123 mmHg, range: 79–139 mmHg. Controlled DBP < 90 mmHg, mean 72.8 mmHg, range: 47–89 mmHg. Uncontrolled SBP ≥ 140 mmHg, mean 155 mmHg, range: 140–217 mmHg. Uncontrolled DBP ≥ 90 mmHg, mean 99.3 mmHg, range: 90–134 mmHg. *Refers to significant differences between males and females.

### 3.1 Gut microbiota composition in the hypertensive older adults

**Figure 1:**
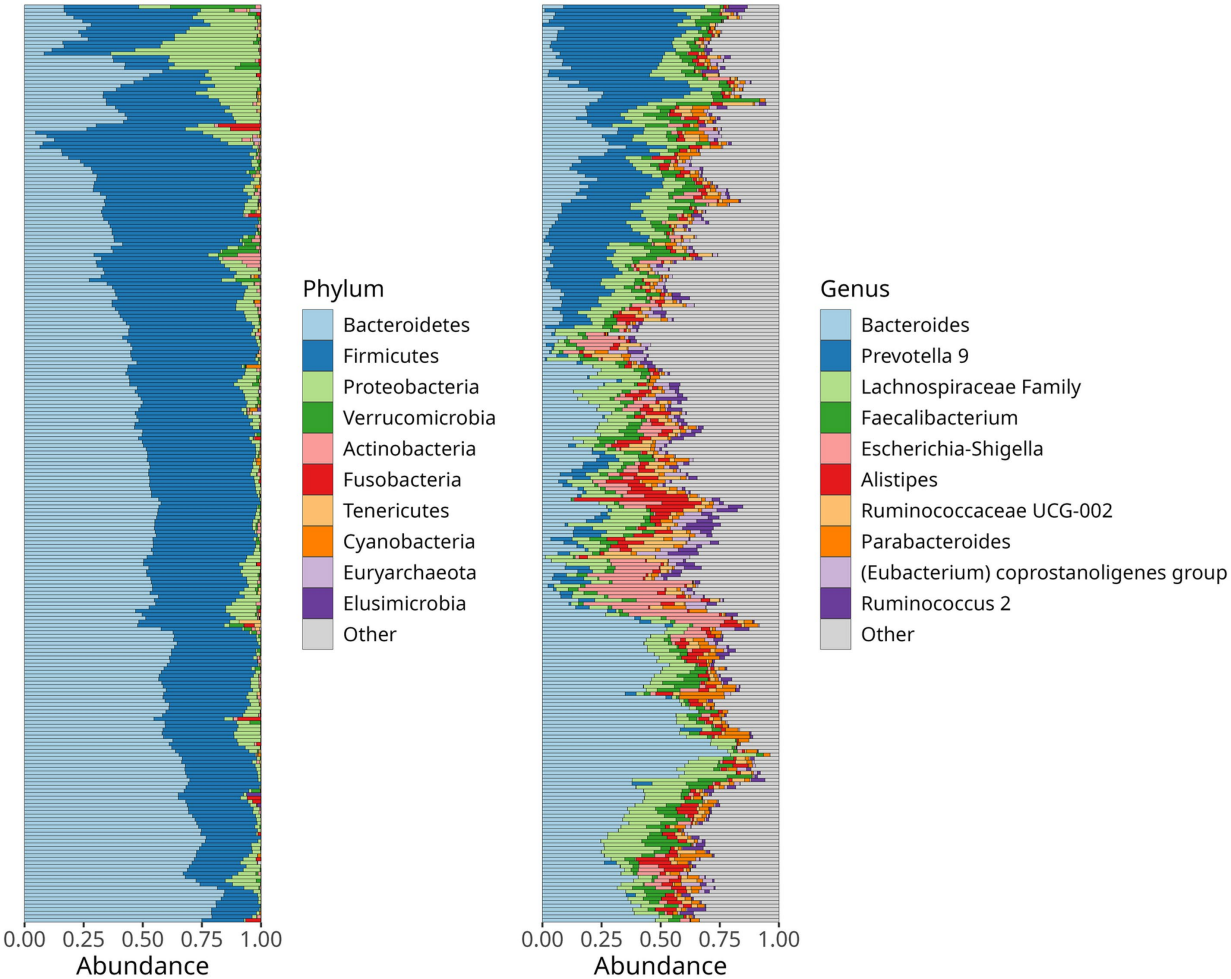
Relative abundance of the dominant bacteria phyla **(A)** and genera **(B)** in the gut microbiome of hypertensive older aldults. Stacked bar charts depict the mean abundance across all samples.

First, we investigated the relative abundance of bacterial phyla and genera, observing that Bacteroidetes showed the highest abundance (49%), followed by Firmicutes (42%), Proteobacteria (7%), and *Verrucomicrobia* (1.0%). At the genus level, *Bacteroides* (27%), *Prevotella 9* (14%), *Faecalibacterium* (5.4%), *Lachnospiraceae*, *Escherichia-Shigella* (4.7%), *Allistipes* (4.2%), *Ruminococcaceae UCG-002* (3.7%), *Parabacteroides* (2.9%), *Eubacterium coprostanoligenes* (2.5%), *Roseburia* (1.8%), and *Christensenellaceae R-7* (1.1%) were the most abundant (Figure 1). No significant differences in microbiota composition according to hypertension control for systolic and diastolic blood pressure (SBP and DBP) were observed (Supplementary Figure 1).

### 3.2 The core microbiome composition in the older adults

**Figure 2:**
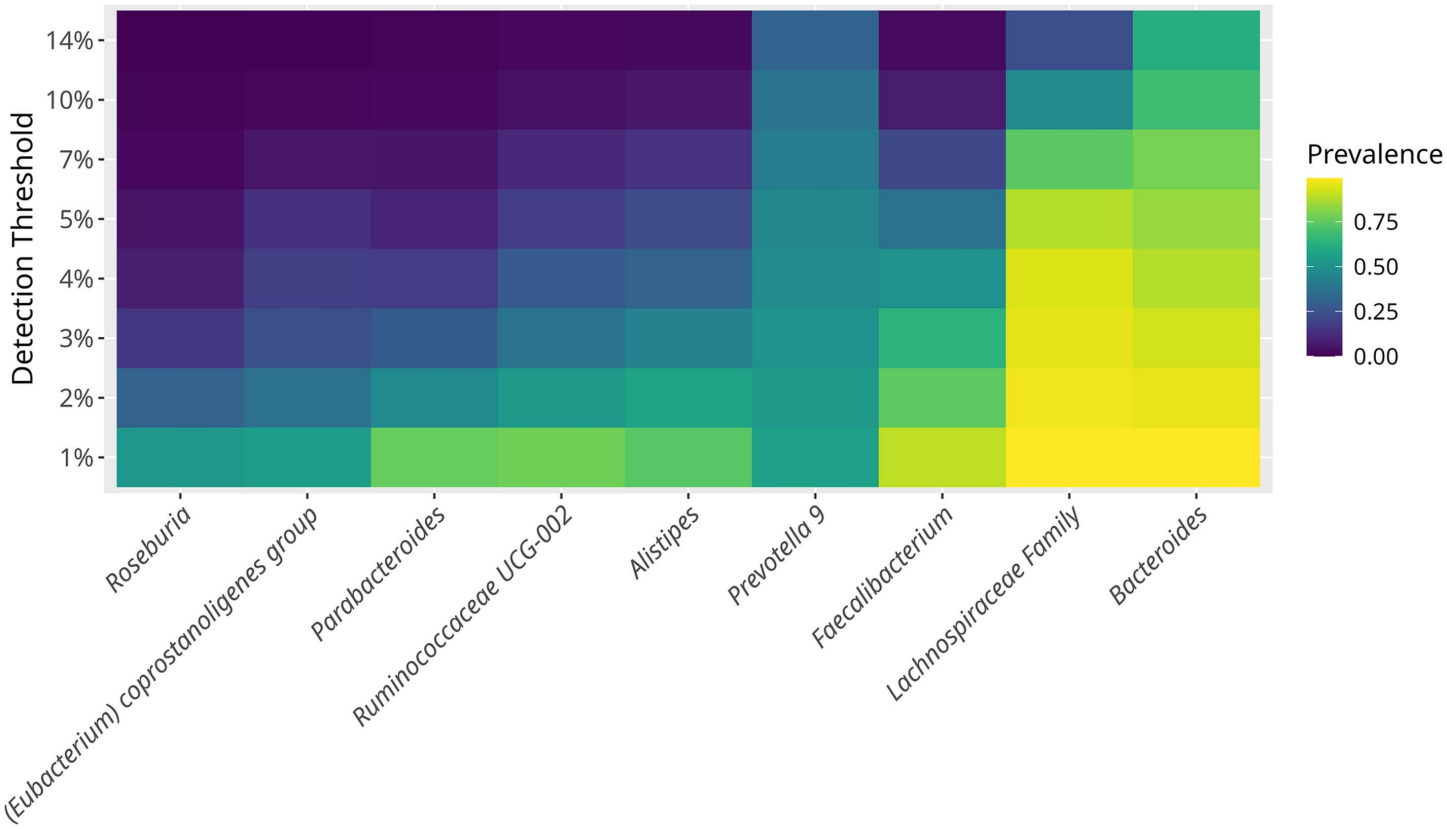
Prevalence of core bacterial taxa in the gut microbiome of hypertensive older adults. The minimum detection thresholds was set at 1%. The maximum prevalence detected was of 14%. Each horizontal line represents a bacterial genus or family. The core microbiome defined as taxa present in >50% of samples at >1% of abundance included *Bacteroides, Prevotella 9, Faecalibacterium, Lachnospiraceae family, Ruminococcaceae UCG-002, Alistipes*, and *Parabacteroides*. The heatmap illustrates how bacterial prevalence changes with increased abundance thresholds.

One of the aims of the study was to characterize the core bacterial composition of the gut microbiome in hypertensive older adults. The prevailing bacteria, ranked by prevalence and abundance, were *Bacteroides, Prevotella 9, Faecalibacterium, Alistipes, Ruminococcaceae UCG-002, Parabacteroides, Eubacterium coprostanoligenes,* and *Roseburia,* showing a prevalence of up to 40% (Figure 2). In addition, bacteria with lower but consistent abundance across the study population included *Escherichia-Shigella, Paraprevotella, Phaseolarctobacterium, Ruminococcus 2, Subdoligranulum, Dialister, Ruminococcaceae UCG-014* & *UCG-005, Ruminococcus 1, Christensenellaceae R-7, Barnesiell*a, and *Blautia,* each with an abundance around 20%.

### 3.3 Alpha diversity

**Figure 3:**
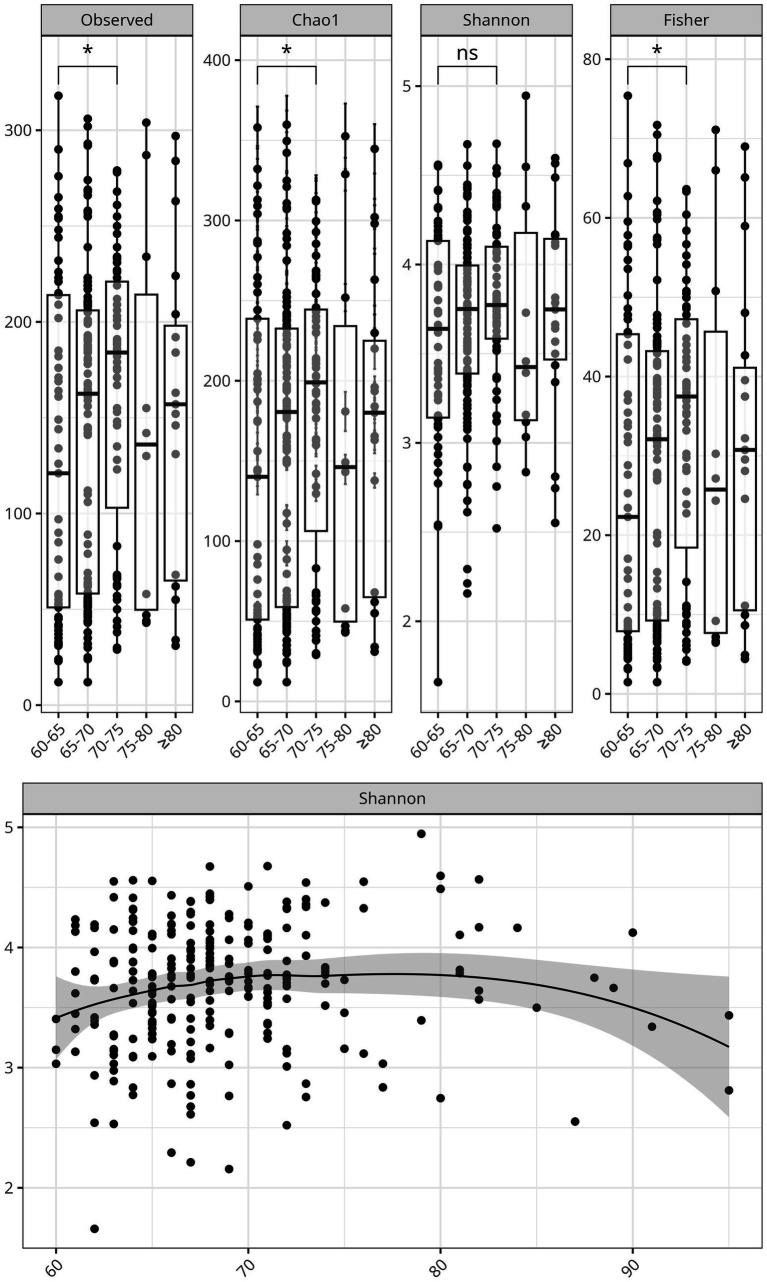
Alpha diversity of the gut microbiome declines with age in hypertensive older adults. **Top:** Boxplots of the alpha diversity indices, Chao1, Observed Species, Shannon, and Fisher stratified by age groups: 60-65, 66-70, 71-80, and >80 years. Significant differences were marked with an asterisk. **Bottom:** Scatter plot showing the negative correlation between Shannon diversity index and age as a continuous variable, with a shaded confidence interval surrounding the regression line.

We evaluated bacterial richness and diversity using several metrics, considering age as a continuous variable, and stratifying by age group. Alpha diversity indexes were compared between the controlled and uncontrolled patients. For the age group 60–75y, the indexes—Chao 1, Fisher, and observed species OTUs—showed higher richness, including more singletons and rare bacteria, compared to the individuals older than 75 years. After 80 years of age, there was an apparent decrease in bacterial richness (Figure 3). Alpha diversity indexes comparing the controlled and uncontrolled SBP and DBP groups did not show significant differences, and these groups seemed similar in terms of abundance and richness. However, there was a lower number of ASVs in the uncontrolled DBP group, as shown by a lower Chao1 index (Supplementary Figure 2).

### 3.4 Beta diversity

**Figure 4:**
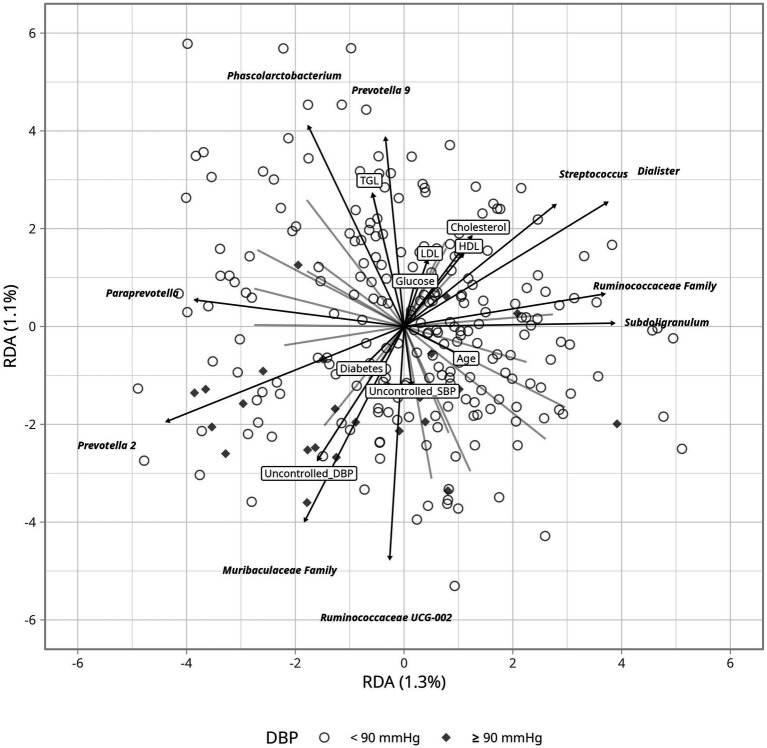
Redundnacy Analysis (RDA) ordination plot depicting the gut microbiome based on DBP and its relation to clinical parameters. Arrows indicate the direction and strenght of the association with clinical variables including glucose levels, cholesterol and blood pressure.

To assess differences in bacterial composition between controlled and uncontrolled hypertension, we estimated a Bray–Curtis dissimilarity index but found no clustering differences when comparing blood pressure levels ≥140/90 mmHg with <140/90 mmHg (Supplementary Figure 3). Hence, we evaluated the impact of clinical variables using RDA, which may explain variation in hypertension control. *Ruminococcaceae* and *Muribaculaceae* seemed to partly accompany uncontrolled hypertension, more apparently for diastolic than for systolic blood pressure (Figure 4). Age was associated with changes in the abundance of *Escherichia-Shigella*, while variations in blood glucose and lipid levels corresponded with changes in *Prevotella 9* and *Phascolarctobacterium* abundance. The correlation analyses between hypertension control and bacteria, including *Escherichia-Shigella,* did not show a relevant relationship despite previous reports (y ≤ 0.02 ~ 0.00002*BP(x), R2 ≤ 0.002, *p* ≥ 0.50).

### 3.5 Bacterial differential abundance according to hypertension control

**Figure 5:**
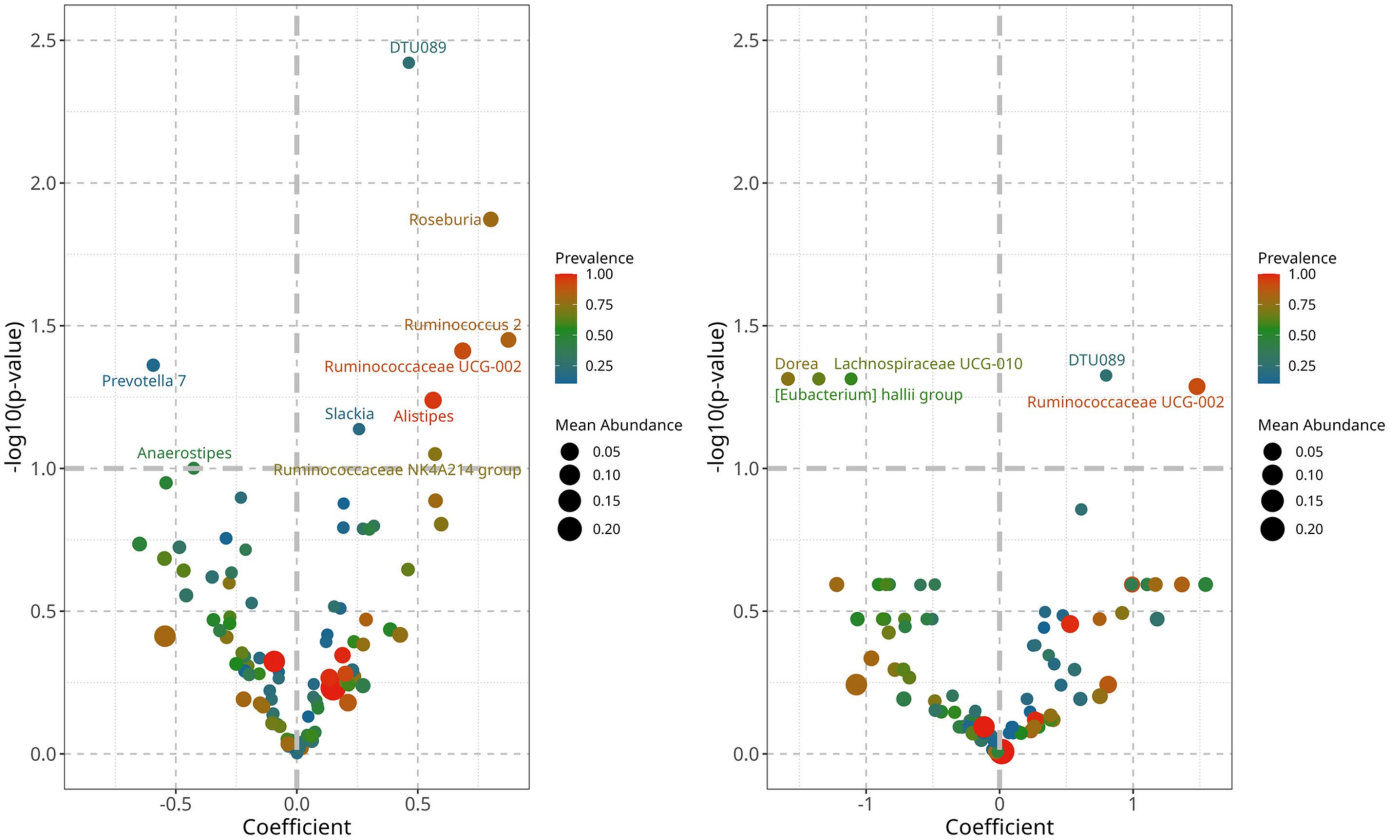
Differential abundance analyisis of gut bacterial genera associated with controlled blood pressure. **Left:** controlled SBP was characterized by an increased abundance of genera, DTU089 and *Ruminococcaceae* UGC-002, and a decrease of *Lachnospiraceae UCG010* and *Eubacterium hallii* group. **Right:** controlled DBP is characterized by an increase of *Roseburia*, DTU089, *Alistipes, Ruminococcaceae UCG-002*, and *Ruminococcus 2*, and a decrease of *Prevotella 7* and *Anaerostipes*.

To identify bacteria that could explain the differences between controlled and uncontrolled hypertensive patients, we performed a linear decomposition analysis (LinDA) and examined associations between the gut microbiome and hypertension control for SBP and DBP separately (Zhou et al., 2022). We found that when hypertension is controlled, there is an increased abundance of *Ruminococcaceae UCG002* and *DTU 089* and a decreased abundance of *Dorea, Lachnospiraceae UCG-010, Eubacterium hallii,* and *Prevotella 7* (Figure 5). Abundance differences were similar in SBP and DBP for *Ruminococcaceae UCG002* and *DTU 089,* but after correction using an FDR test, statistical significance was observed only for DBP.

### 3.6 Functional analysis predictions for hypertension control

Functional analyses of metagenomic predictions based on bacterial abundance differences between controlled and uncontrolled hypertension may provide information on macromolecules and their routes, potentially further explaining the relationship between the gut microbiome and hypertension control. We observed three major gene-enzyme differential changes: (a) a predicted increase in genes and enzymes related to the production of reactive oxygen species in the uncontrolled patients, including, EC1.6.3.3, K17870, and K180022; (b) a decrease in predicted genes and enzymes related to nucleic acid metabolism, including, EC2.1.1.215., K18846, K00555, and K19174/75; and (c) an increase in predicted genes and enzymes related to the biotransformation of endogenous and xenobiotic compounds, including conjugation, transferases, and multidrug-resistant enzymes and genes such as EC2.4.1.19, EC2.1.1.180, EC2.1.1.215, K18908, K15546, and K00701 (Supplementary Table 1).

## 4 Discussion

Cardiovascular disease is the leading cause of death worldwide, with hypertension and its complications accounting for over 50% of these deaths, according to the World Health Organization. Research in the field has become multidisciplinary to better tackle the diversity of metabolic paths involved in hypertension. Consequently, research focused on the gut microbiome has shown some revealing differences in the gut bacteria of hypertensive individuals compared to normotensive individuals, providing hypotheses that support the role of bacteria as probiotics or enhancers of drug efficacy (Dinakis et al., 2022; Verhaar et al., 2020). Here, we characterized the core gut microbiome of 240 urban hypertensive older adults and investigated if hypertension control could be explained in part by microbial abundance differences. We discussed our findings in the context of current knowledge.

### 4.1 The core microbiome of the older adults

The composition of the gut microbiome in older adults is highly variable, yet certain patterns have been consistently reported. For instance, Bacteroidetes and Firmicutes dominate 80% of the gut microbiome, with an increase in Proteobacteria and *Escherichia-Shigella* with age (Novelle et al., 2025; Salazar et al., 2017). Our findings align with previous observations, as we observed that Firmicutes and Bacteroidetes together comprise up to 91% of the gut microbiota, with Bacteroidetes being more prevalent (49%) than Firmicutes (41%; Bradley and Haran, 2024).

The core bacteria belonged to the Bacteroidetes phylum, including *Bacteroides, Parabacteroides, Prevotella 9, and Alistipes,* as well as to the Firmicutes phylum, including *Faecalibacterium, Ruminococcaceae UCG-002, Eubacterium coprostanoligenes,* and *Roseburia,* together composing up to 40% of the gut microbiome (Figure 2). This is consistent with previous reports on the gut microbiome of older adults (O’Toole and Claesson, 2010).

Several of the above-mentioned bacteria have been associated with aging. For example, *E. coprostanoligenes* has been detected in older adults, and its abundance increases with age and has been observed in the transition from adulthood to older age (Biagi et al., 2010; Sepp et al., 2022; Wei et al., 2021). Hence, the presence of *E. coprostanoligenes* was not surprising and could be related to the age of this population. The presence of *Bacteroides, Parabacteroides, Alistipes*, and *Prevotella 9* characterizing the gut microbiome of the older adults in this study further confirms their role as major components of the gut microbiome, as reported in previous studies (Biagi et al., 2012; O’Toole and Claesson, 2010). Potential roles for each genus have been discussed before. *Prevotella 9* is associated with accelerated aging and the inflammaging phenotype (Singh et al., 2024). The role of *Parabacteroides* in older adults is inconclusive. Some studies have found it to be more abundant in patients with Alzheimer’s disease, while others have associated it with positive outcomes related to mental health and diet. Its abundance seems to accompany that of several strains of *Ruminococcacea*e, which was also observed here (Molinero et al., 2023; Olson et al., 2018)*. Alistipes*, another age-dependent bacterium, has been associated with anti-inflammatory properties, SCFA production, and beneficial gut health. Nevertheless, its overabundance has been reported in inflammatory bowel disease and hypertension, highlighting the fact that its roles are not fully clear and cannot be interpreted in isolation (Tokarek et al., 2023).

Furthermore, in characterizing the microbiome of hypertensive older adults, we observed several bacteria with an average abundance of 20%, including *Ruminococcaceae*, *Phascolarctobacterium Subdoligranulum, Dialister, Blautia, Barnesiell*a, *Paraprevotella, Christensenellaceae R-7,* and *Escherichia-Shigella,* all of which have been reported to increase with age (Farsijani et al., 2024; M et al., 2022). The presence of *Escherichia-Shigella* is causative of the “inflammaging” phenotype, possibly mediated by its glycerophospholipid metabolism, which activates toll-like receptors, increasing inflammation and endothelial dysfunction (Wang et al., 2021). It is well documented that both *Escherichia-Shigella* and hypertension risk increase with age (Mushtaq et al., 2019). While in this study, we observed a higher abundance of *Escherichia-Shigella* with age, we did not observe a relationship between *Escherichia-Shigella* and increased blood pressure. This discrepancy may be due to age-related microbial dynamics. Our study population consisted predominantly of individuals aged 70-80 years, while most research documenting the increase of *Escherichia-Shigella* in hypertension tends to include a broader age range and younger individuals (Wan et al., 2021). It is possible that age itself (via inflammaging) may override or mask the distinctions based on hypertension control status. *Escherichia-Shigella* may already be elevated due to age in both groups, limiting our ability to detect differences associated with blood pressure control.

### 4.2 Alpha diversity

Variation in gut microbial diversity with age and hypertension control was analyzed using several metrics. Consistent with current research, we observed a decline in gut bacterial diversity with age (Renson et al., 2020), and a clearer trend was observed in individuals aged 75 y and older (Figure 3). Interestingly, the 65-70y age group displayed increased diversity, which persisted up to age 75. This suggests that the transition between 65 years and 70 years could represent a window for enhancing and protecting microbial diversity before its age-related decline. The uncontrolled DBP group showed lower diversity compared to the controlled DBP group (*p* < 0.05) for the Chao 1 index, supporting the notion that healthier phenotypes show a richer gut microbiota (Louca et al., 2021).

### 4.3 Beta diversity

Several metrics were assessed to investigate microbial differences according to hypertension control. Average bacterial composition was not different between the controlled and uncontrolled patients, but *Ruminococaceae UCG-002* and the *Muribaculaceae* family were able to explain variation in uncontrolled hypertension when considering clinical parameters (Figure 4). *Ruminococcaceae UCG-002* has been negatively associated with heart disease (Li et al., 2022) and metabolic syndrome (Wutthi-in et al., 2020). The beneficial effects of the *Ruminococcaceae* family are related to the production of SCFAs, which modulate blood pressure through the kidney receptors—GPR41, GPR43, and GPR109A (Gao et al., 2021; Tilves et al., 2022). *Ruminocococcus* can also improve linoleic acid and glucose absorption, insulin sensitivity, and intestinal integrity in mice, which together may support cardiovascular health (Xie et al., 2022). *Muribaculaceae* abundance seems to accompany uncontrolled DBP (Figure 4). This family has been associated with both anti-inflammatory and pro-inflammatory effects depending on the environment (Zhu et al., 2024). Therefore, its role in uncontrolled DBP would require targeted experiments.

### 4.4 Bacterial differential abundance characterizing hypertension control

We found interesting differences in bacteria associated with controlled blood pressure that have not been previously acknowledged. For example, increases in *Ruminococcaceae UCG002, Ruminococcus 2,* and *DTU 089—*the latter being a member of the *Lachnospiraceae* family—have been associated with skeletal metabolism and low protein intake in older adults (Farsijani et al., 2022; Okoro et al., 2023). In this study, we validated the presence of *DTU 089* in older adults, inferring that its potential influence on health could be related to diet. *Ruminococcaceae UCG002* has been reported to negatively influence cardiovascular health and hypertension (Chen et al., 2022; Miao et al., 2024; Qin et al., 2022). However, most members of the *Ruminococcaceae family* ferment fiber to produce SCFAs and influence gut integrity, immunity, and cholesterol transport. Therefore, future research should focus on specific species and strains to elucidate their clear role (Sanders et al., 2019; Vojinovic et al., 2019).

On the other hand, controlled blood pressure was associated with decreased abundance of *Dorea, Prevotella 7, Lachnospiraceae UCG010, and the Eubacterium hallii* group (Figure 5). Except for *Prevotella 7,* these bacteria belong to the phylum Firmicutes and the family *Lachnospiraceae*, and members of this family can show contrasting roles in health and disease (Vacca et al., 2020). *Dorea* has been associated with hypertension and its complications (Miao et al., 2024), and *Lachnospiraceae UCG10* has been linked as a causal factor in venous thromboembolism (Huang et al., 2025). *Eubacterium hallii,* also known as *Anaerobutyricum hallii,* is consistently reported as beneficial, since it can produce butyrate, reuterin, and vitamin B12 (Engels et al., 2016). It has also been suggested as a probiotic for cancer prevention, as it can conjugate carcinogenic pyrimidine derivatives from cooked meat (Engels et al., 2016). The beneficial properties of these members of the *Lachnospiraceae* family are consistent with their decreased abundance in a hypertensive population. Investigations should decipher metabolic pathway differences within family members so that specific *Lachnospiraceae* species can be pinpointed as more sensitive or beneficial for blood pressure control and to clarify if these associations could lead to hypertension development or protection pathways. Of special interest is the *Enterobacter hallii group,* whose abundance has been associated with age (Biagi et al., 2010), hypertension (Verhaar et al., 2020), inflammation, and insulin sensitivity (Udayappan et al., 2016). Its decrease in hypertensive older adults may reflect a direct depletion of its beneficial activity. However, the directionality of its abundance in healthy older adults, as well as the impact of medication and different geographical ancestries, remains unclear. Therefore, further research is needed in this area (Hayashi et al., 2003; Mueller et al., 2006).

Finally, functional predictions indicated that uncontrolled patients may have an increased molecular machinery for the generation of reactive oxygen species and metabolizing enzymes, along with a decrease in nucleic acid metabolism or repair. These observations are broad and do not directly reflect the gut functional environment. We can only infer that higher reactive oxygen species and poor nucleic acid maintenance are consistent with the phenotype of uncontrolled hypertensive patients. It is interesting to note that functional predictions also suggested an increase in metabolizing enzymes, some of which could be involved in phase II drug metabolism. This indicates that the gut microbiome of uncontrolled patients may be metabolizing drugs, such as hypertensives, at a higher rate, likely affecting their efficacy.

In summary, changes in the abundance of *Ruminococcus UCG-002* and several members of the *Lachnospiraceae* family, such as *Dorea, Eubacterium hallii,* and *Lachnospiraceae UCG-010,* were significantly associated with hypertension control. These associations suggest that members of the *Lachnospiraceae* family may contribute to the regulation of blood pressure through fiber fermentation and SCFA production, followed by interactions with kidney receptors, renin, and lipopolysaccharides (Verhaar et al., 2020; Yan et al., 2022). In addition, these bacteria produce metabolites such as dopamine, serotonin, and norepinephrine, which favor angiotensin II release and vasoconstriction (Miao et al., 2024; Xu and Marques, 2022). It is relevant to mention that this study included only hypertensive individuals and did not include a normotensive reference, which may reflect a limitation of the study. Consequently, the directionality of the observed abundance changes may not fully align with previous studies.

Nevertheless, we depicted novel observations that, on the one hand, described the core microbiome of hypertensive older urban dwellers, confirming and expanding previous associations between blood pressure and SCFA producers such as *Dorea, Ruminococcaceae UCG-01,0,* and the *Lachnospiraceae family*. On the other hand, we conducted one of the largest studies in an underexplored admixed population, showing an association between gut bacteria and hypertension control.

These observations are among the first to describe the gut microbiome of hypertensive older adults, contributing to the growing body of evidence on the role of the microbiome and its changes in aging and hypertension. Future endeavors should include a normotensive group, as well as assessments of lifestyle and dietary habits, to broaden and validate the observed changes in the gut microbiome of hypertensive older adults.

## Data Availability

The original contributions presented in the study are publicly available. This data can be found here: https://www.ncbi.nlm.nih.gov/, accession number PRJNA1247541.
