# Supplementary material for: Changes in the gut microbiome of older adults according to hypertension control
Source: Front Microbiol. 2025 Sep 2;16:1605271. doi: 10.3389/fmicb.2025.1605271 (PMC12438838; doi:10.3389/fmicb.2025.1605271)
Supplement: Supplementary file 1 [file Data_Sheet_1.PDF]

Supplementary Material

1.1 Supplementary Material

The following supporting information is included for this manuscript. Supplemental Figure S1: Relative abundance of the gut microbiota according to hypertension control; Supplemental Figure S2: Alpha diversity according to hypertension control; Supplemental Figure S3: Beta diversity according to hypertension control (Bray-Curtis).

Supplemental Figure 1. Relative abundance of the gut microbiota in controlled (top) vs. uncontrolled (bottom) hypertensive patients.

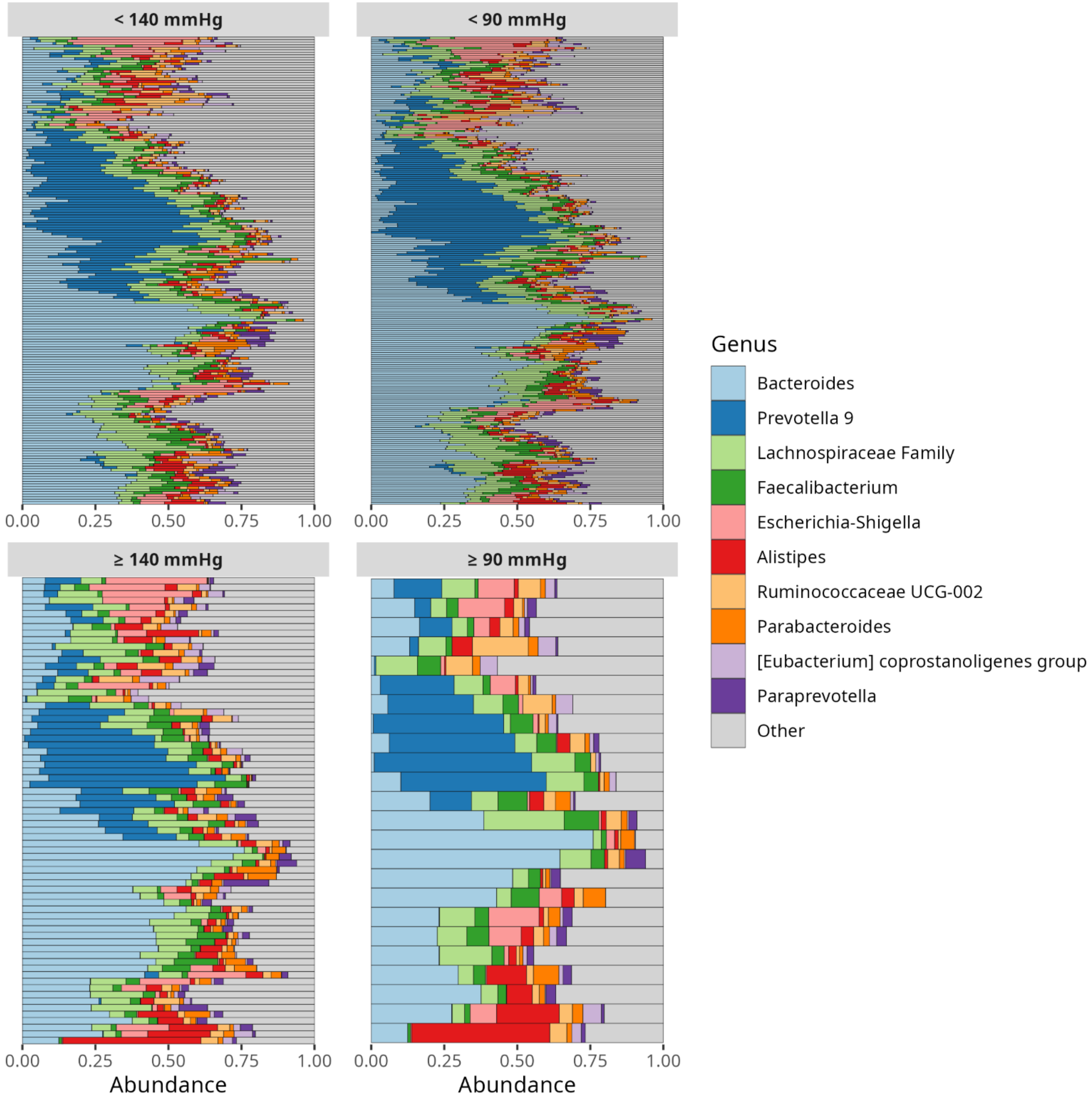

Figure S2: Alpha diversity according to hypertension control;

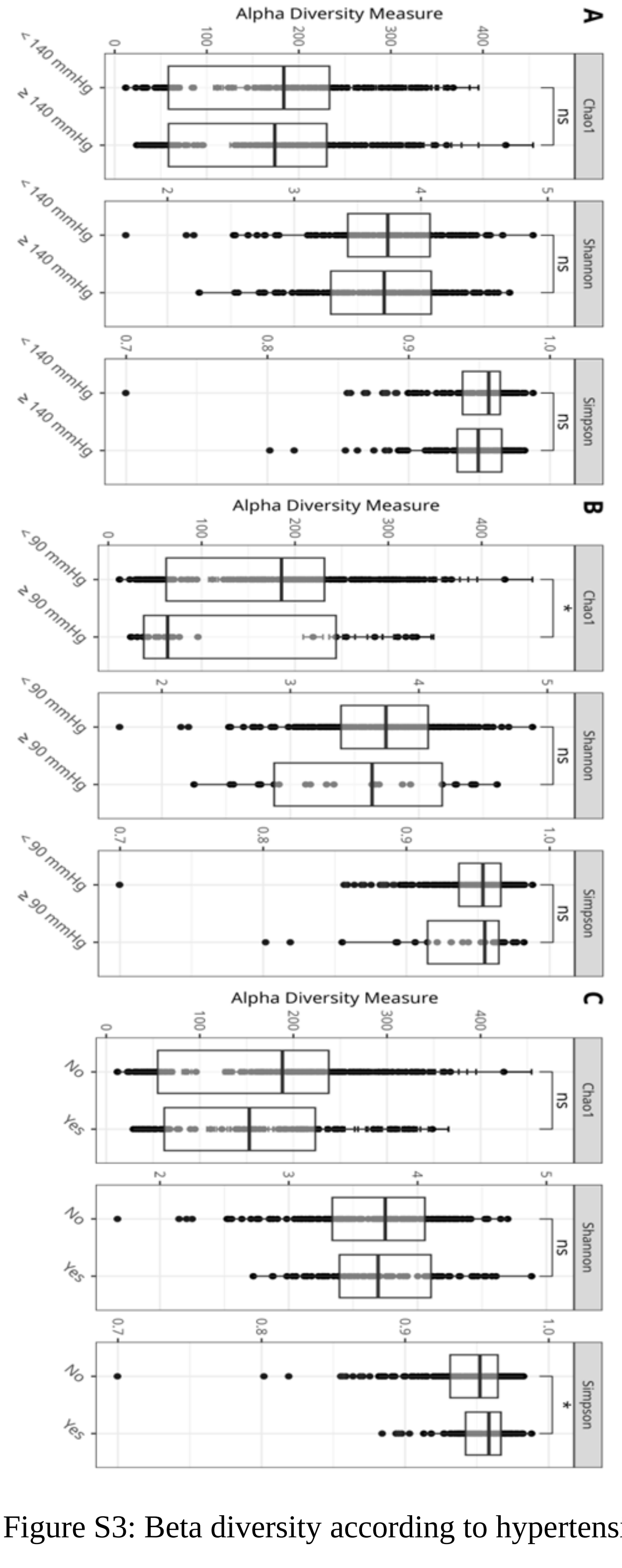

Figure S3: Beta diversity according to hypertension control (Bray-Curtis)

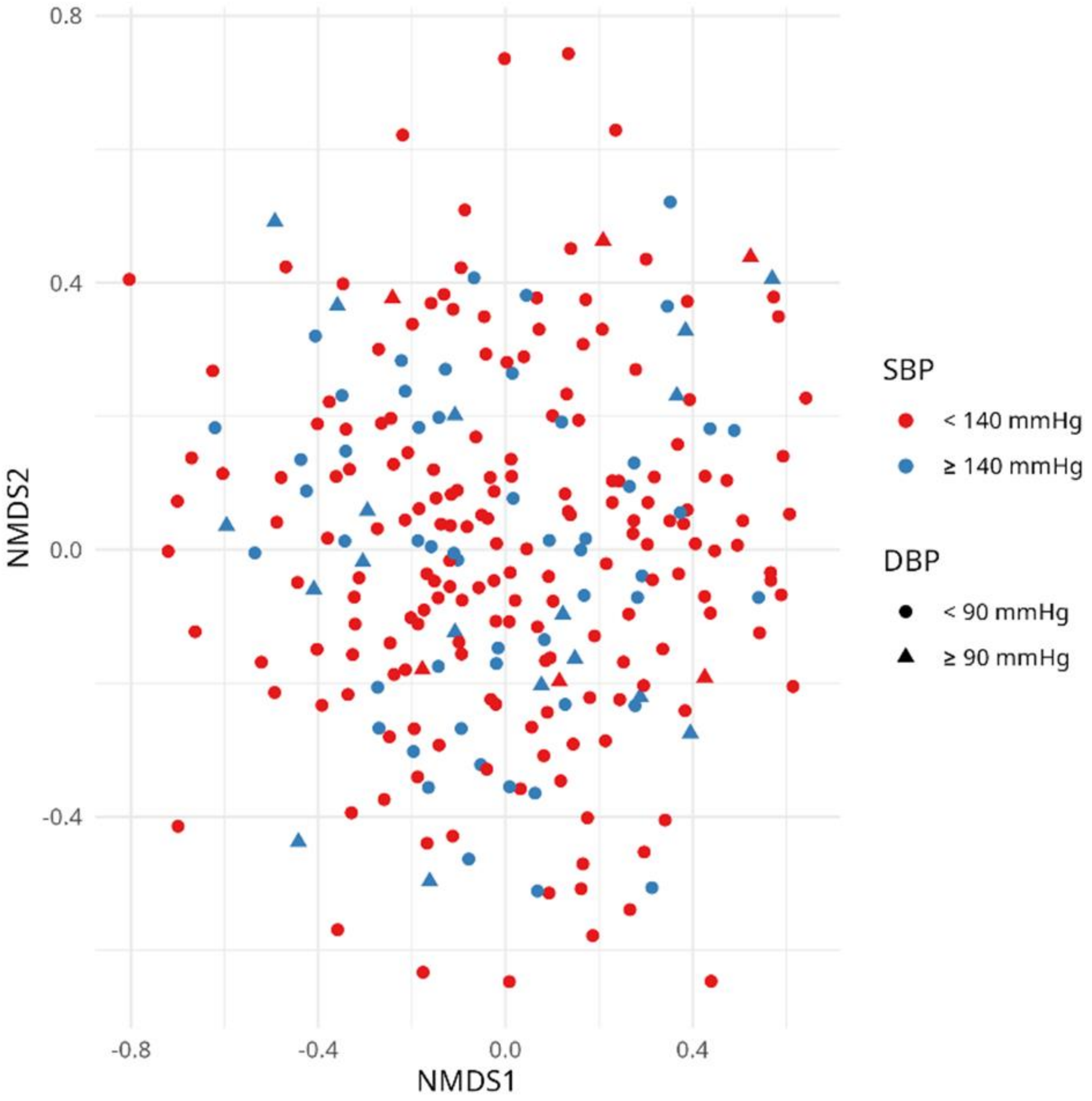
0.8
0.4
0.0
-0.4
NMDS2
-0.8
-0.4
0.0
0.4
NMDS1
SBP
< 140 mmHg
≥ 140 mmHg
DBP
< 90 mmHg
≥ 90 mmHg
