# Supplementary material for: Changes in the gut microbiome of older adults according to hypertension control
Source: Front Microbiol. 2025 Sep 2;16:1605271. doi: 10.3389/fmicb.2025.1605271 (PMC12438838; doi:10.3389/fmicb.2025.1605271)
Supplement: Supplementary file 2 [file Data_Sheet_2.PDF]

ST1. Functional analysis of genes and enzymes differential abundance in uncontrolled versus controlled hypertension

DESeq of predicted enzimes. Diastolic Blood Pressure. Uncontrolled vs controlled (Reference)

baseMean	log2FC	lfcSE	padj	EC	Description
28,76	2,62	0,56	0,00	EC:1.6.3.3	NADH oxidase (H2O2-forming)
11,52	-1,77	0,44	0,04	EC:2.1.1.180	adenosine(1408) in 16S rRNA + S-adenosyl-L-methionine = N(1)-methyladenosine(1408) in 16S rRNA + S-adenosyl-L-homocysteine + H(+)
26,77	2,76	0,70	0,04	EC:2.1.1.215	tRNA (guanine(26)-N(2)/guanine(27)-N(2))-dimethyltransferase
26,77	2,76	0,70	0,04	EC:2.1.1.216	tRNA (guanine(26)-N(2))-dimethyltransferase

DESeq of predicted enzimes. Systolic Blood Pressure. Uncontrolled vs controlled (Reference)

baseMean	log2FC	lfcSE	padj	EC	Description
67,72	-1,78	0,39	0,01	EC:3.4.17.21	glutamate carboxypeptidase II
73,86	-1,66	0,40	0,02	EC:2.4.1.19	cyclomaltodextrin glucanotransferase
10,52	-1,30	0,32	0,03	EC:3.2.1.132	Endohydrolysis of beta-(1->4)-linkages between D-glucosamine residues in a partly acetylated chitosan

DESeq of predicted KO. Diastolic Blood Pressure. Uncontrolled vs controlled (Reference)

baseMean	log2FC	lfcSE	padj	KO	Description
26,31	2,81	0,70	0,03	K00555	K00555 TRMT1, trm1; tRNA (guanine26-N2/guanine27-N2)-dimethyltransferase [EC:2.1.1.215 2.1.1.216]
62,92	-3,26	0,75	0,01	K10105	K10105 LIPT1; lipoyltransferase 1
0,98	1,14	0,27	0,02	K11493	K11493 RCC1; regulator of chromosome condensation
12,75	1,84	0,41	0,01	K14274	K14274 xylC; xylono-1,5-lactonase [EC:3.1.1.110]
7,68	2,79	0,66	0,02	K15546	K15546 boxR, bzdR; XRE family transcriptional regulator, aerobic/anaerobic benzoate catabolism transcriptional regulator
28,28	2,65	0,56	0,01	K17870	K17870 nox1; NADH oxidase (H2O2-forming) [EC:1.6.3.3]
63,07	-3,20	0,73	0,01	K18022	K18022 cutC; glyceraldehyde dehydrogenase small subunit [EC:1.2.99.8]
11,39	-1,77	0,44	0,03	K18846	K18846 npmA; 16S rRNA (adenine(1408)-N(1))-methyltransferase [EC:2.1.1.180]
63,87	-3,24	0,73	0,01	K18908	K18908 mepA; MATE family, multidrug efflux pump
68,60	2,05	0,46	0,01	K19360	K19360 NPHP3; nephrocystin-3
21,44	-5,98	1,47	0,03	K19551	K19551 pelC; pectate lyase C [EC:4.2.2.2 4.2.2.10]

DESeq of predicted KO. Systolic Blood Pressure. Uncontrolled vs controlled (Reference)					
baseMean	**log2FC**	**lfcSE**	**padj**	**KO**	**Description**
853,76	0,88	0,20	0,02	K00082	K00082 ribD2; 5-amino-6-(5-phosphoribosylamino)uracil reductase [EC:1.1.1.193]
199,00	-1,55	0,38	0,02	K00446	K00446 dmpB, xylE; catechol 2,3-dioxygenase [EC:1.13.11.2]
73,97	-1,66	0,40	0,02	K00701	K00701 cgt; cyclomaltodextrin glucanotransferase [EC:2.4.1.19]
10,39	-1,28	0,31	0,03	K01233	K01233 csn; chitosanase [EC:3.2.1.132]
33,48	2,91	0,61	0,00	K02251	K02251 comQ; competence protein ComQ
17,50	5,34	1,11	0,00	K03120	K03120 TBP, tbp; transcription initiation factor TFIID TATA-box-binding protein
24,21	4,95	0,81	0,00	K07463	K07463 K07463; archaea-specific RecJ-like exonuclease
93,60	-2,08	0,48	0,02	K10909	K10909 luxQ; two-component system, autoinducer 2 sensor kinase/phosphatase LuxQ [EC:2.7.13.3 3.1.3.-]
79,28	-2,80	0,71	0,04	K10948	K10948 hlyA; hemolysin
17,59	2,40	0,50	0,00	K14192	K14192 clfB; clumping factor B
0,42	8,85	1,89	0,00	K14197	K14197 sbi; immunoglobulin G-binding protein Sbi
10,75	1,14	0,29	0,04	K17870	K17870 nox1; NADH oxidase (H2O2-forming) [EC:1.6.3.3]
74,46	-3,60	0,86	0,02	K18454	K18454 deaA; chitin disaccharide deacetylase [EC:3.5.1.105]
62,70	-1,30	0,30	0,02	K19174	K19174 dptG; DNA phosphorothioation-dependent restriction protein DptG
49,32	-1,25	0,29	0,02	K19175	K19175 dptH; DNA phosphorothioation-dependent restriction protein DptH
93,46	-2,09	0,50	0,02	K19693	K19693 tfoS; AraC family transcriptional regulator, chitin signaling transcriptional activator

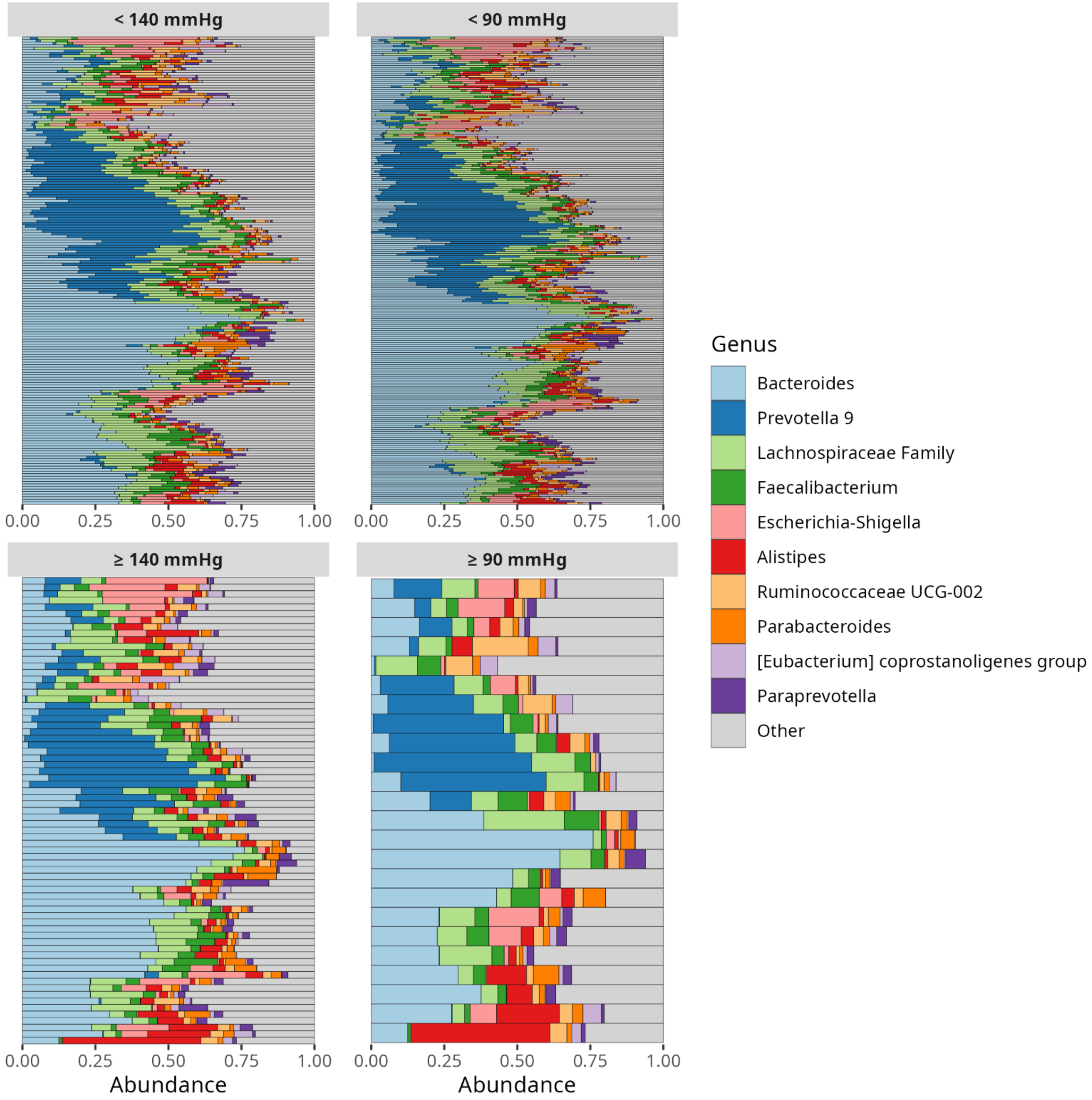

Figure S2: Alpha diversity according to hypertension control;

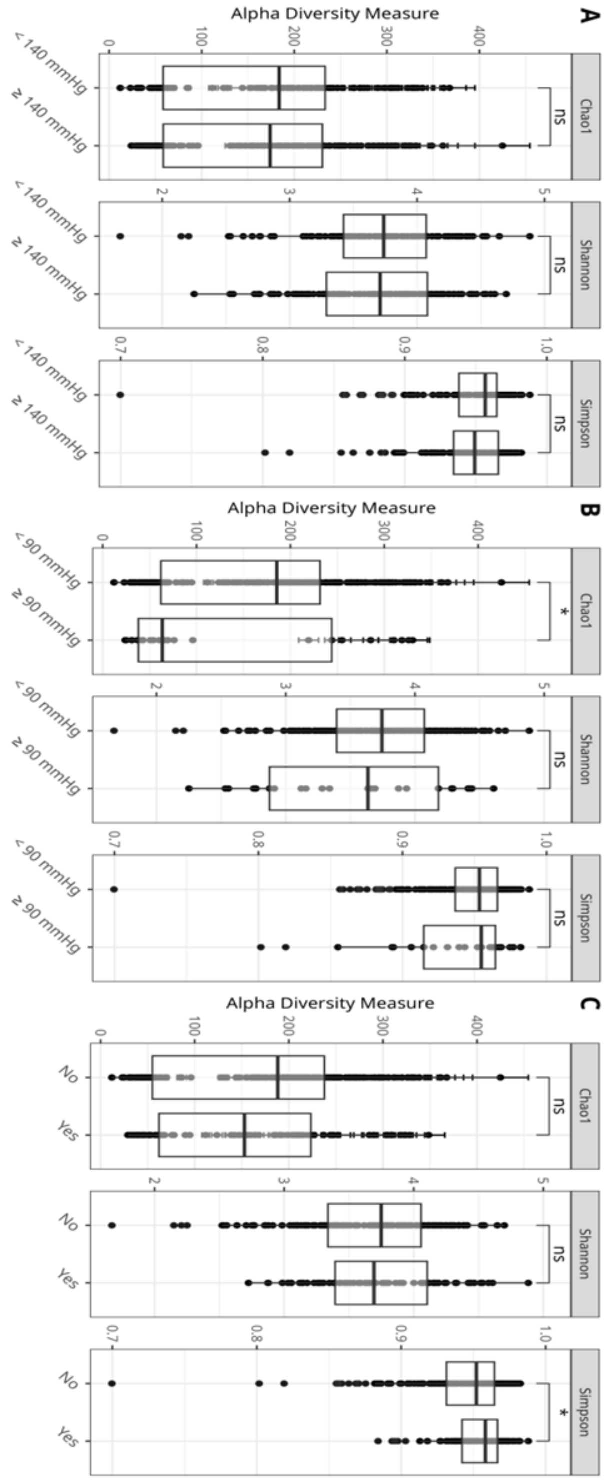

Figure S3: Beta diversity according to hypertension control (Bray-Curtis)

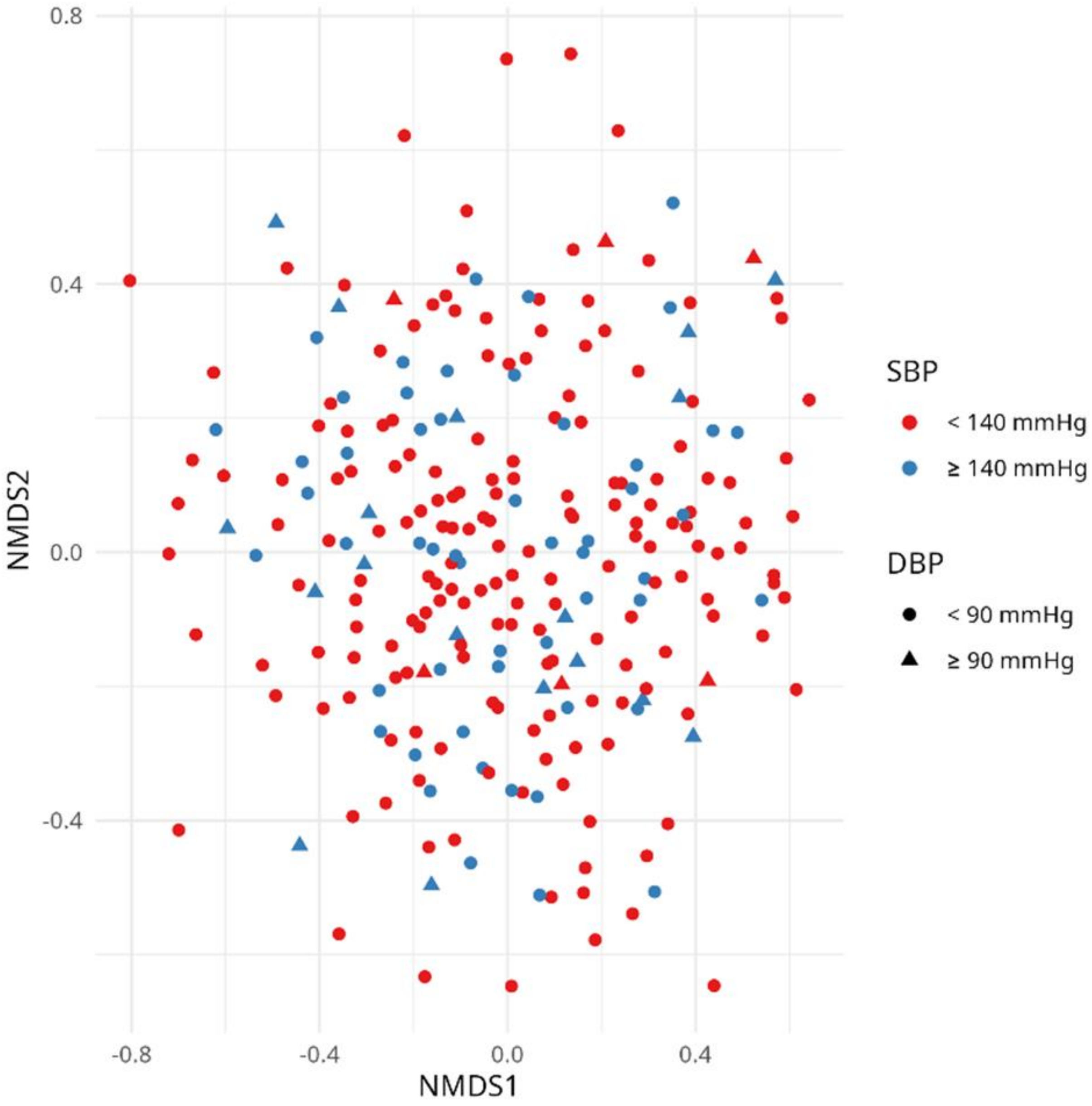
0.8
0.4
0.0
-0.4
NMDS2
-0.8
-0.4
0.0
0.4
NMDS1
SBP
< 140 mmHg
≥ 140 mmHg
DBP
< 90 mmHg
≥ 90 mmHg
